# Prussian Regulations Regarding Leeches

**Published:** 1838-10

**Authors:** 


### PRUSSIAN REGULATIONS REGARDING LEECHES.

The growing scarcity of leeches in Germany has led to a large importation of Hungarian leeches into that country. The German and Hungarian leech, although belonging to the same genus, are of different species. The back of the former *Sanguisuga medicinalis,* Savigny,) is of an olive green, and has six rust-coloured longitudinal strips, generally dotted with black; and the belly is of a greenish yellow, spotted black. The back of the Hungarian leech (*Sanguisuga officinalis,* Savigny,) is green, or dark green, with six longitudinal rust-coloured strips; and the belly is olive green, and not spotted.

It was the general opinion that the Hungarian leech was inferior to the German leech in energy; but, in order to determine the point with certainty, exact experiments were instituted at the Charité hospital, from which it appeared that one German leech was nearly equal to two Hungarian. The great difference in the size of leeches also attracted the attention of the experimenters; and the Prussian government has, in consequence, published an ordinance, by which apothecaries are bound to divide their leeches into three classes:

1. *Sanguis ponderis minimi,* or leeches not exceeding thirty grains in weight.
2. *S. pond. med.,* or leeches weighing between thirty and sixty grains.
7. *S. pond. maxim.,* or leeches weighing between sixty and ninety grains.

Leeches above ninety grains in weight are not to be used in medicine, unless expressly ordered; and practitioners are required to state in their prescriptions the size and species of the leeches wanted. *Medicinisch Zeitung.* No. 2. 1838.

